# From education to employment: BioSci Toolkit CIC’s unique approach to EDI, access and inclusive scientific careers

**DOI:** 10.1042/ETLS20253028

**Published:** 2025-12-23

**Authors:** Chinedu Agwu

**Affiliations:** 1 Brunel University of London, London, U.K.

**Keywords:** bioscience workforce, outreach initiatives, STEM talent pipeline, structural inequities, UK life sciences sector

## Abstract

The UK life sciences sector, valued at approximately £100 billion and employing nearly 300,000 individuals, is positioned for transformative growth under the government’s ten-year Life Sciences Sector Plan. Despite significant investment in R&D, clinical trials and health data infrastructure, structural inequities persist within the science, technology, engineering and maths talent pipeline, limiting the participation and progression of individuals from marginalised and underrepresented groups. Historical and systemic biases have contributed to enduring underrepresentation in leadership, academia and industry, constraining innovation and weakening public trust in science. BioSci Toolkit CIC addresses these challenges through targeted mentorship, skills development and outreach initiatives that enhance confidence, competence and professional networks for underrepresented students and early-career scientists. By providing visibility, guidance and pathways into vocational and academic opportunities, the initiative seeks to strengthen diversity, equity and inclusion in UK bioscience, ensuring that the workforce better reflects societal demographics while fostering innovation and societal impact.

## A fast-moving UK life sciences landscape

The UK life sciences sector—valued at around £100 billion and employing approximately 300,000 people—has never been more dynamic [1]. The government’s recent Life Sciences Sector Plan lays out a ten-year roadmap to translate research into real-world healthcare outcomes, with over £2 billion in public funding directed toward R&D, health data infrastructure and clinical trial acceleration [1]. Yet challenges loom—UK life sciences risk losing out by £15 billion annually compared with global peers, underscoring the urgency of building an inclusive, resilient pipeline of skilled workers [2]. As of 2023, approximately 9.4 million people were employed in jobs related to science, technology, engineering and maths (STEM) in the UK. The UK STEM sector is considered important by many for future economic growth [3]. There are concerns around the availability of STEM skills in the UK workforce; 49% of engineering and technology businesses report difficulties with recruitment because of skills shortages [3].

For many years, science has been constructed and perceived as the domain of white, non-disabled, affluent men—a narrative that continues to be perpetuated through the persistent underrepresentation of diverse role models in senior leadership positions and in public depictions of scientists [4]. Unconscious bias, systemic discrimination and the lack of representation function together to reinforce and reproduce the persistence of this stereotype [4]. The exclusion of diverse scientists from leadership, academia and media not only reflects existing inequities but also perpetuates them by constraining the visibility of role models, thereby shaping aspirations and limiting pathways for women and individuals from marginalised groups [5,6]. This cycle of exclusion is particularly pronounced for individuals who lack familial or community support, as the absence of such social resources diminishes access to encouragement, mentorship and networks that are often critical for navigating barriers to entry. Without these forms of support, structural inequities are intensified, further constraining opportunities and discouraging the pursuit of STEM careers [6]. This alludes to the concept of intersectionality, which highlights how gender and race interact as markers of structural inequality. Over time, this approach has been expanded to include additional dimensions, such as class, disability and sexual orientation. A better understanding of this will help to promote equity in the scientific workforce [7–10]. Finally, the lack of diversity in science can have cascading effects that both constrain innovation and undermine public trust. Limited representation narrows the perspectives and approaches that drive scientific discovery, while simultaneously reinforcing a disconnect between scientific institutions and the diverse communities they are intended to serve [11].

In response to these persistent inequities, BioSci Toolkit CIC provides a timely, grassroots initiative, empowering underrepresented students and early-career scientists with the skills, confidence and professional networks necessary to succeed and contribute meaningfully to the life sciences.

## About BioSci Toolkit CIC – mission and vision

Founded to democratise access to bioscience careers, BioSci Toolkit Community Interest Company (CIC) combines mentorship, soft-skill workshops and networking opportunities to support students and recent graduates. Its mission is clear: to empower students, especially from Black, mixed and seldom-heard ethnic communities, to confidently make informed decisions about bioscience degrees and careers, by nurturing confidence as much as competence. They envision a world where students from Black, mixed and seldom heard communities are fully represented and thriving in bioscience. They plan to become a leading force for equity in bioscience offering tools, networks and guidance from school classroom to career. They primarily focus on tackling barriers associated with ethnicity and socioeconomic status and currently operate in London, England, with plans to scale nationally. Table 1 provides a detailed overview of the mission and services offered.

**Table 1:** A summary of BioSci Toolkit deliverables.

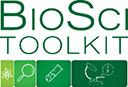 **BioSci toolkit**	
**Origin**	Launched in May 2023
**Mission**	Exists to empower students—especially from Black, mixed and seldom-heard ethnic communities—to confidently make informed decisions about bioscience degrees and careers.
**Vision**	We plan to become a leading force for equity in bioscience
**Services**	Educational resources e.g. lab report template Online skills workshops e.g. lab report writing, study skills etc. School visits for college students (age 16–19) BioSci Future Leaders Mentorship Scheme BioSci Launchpad Work Experience Scheme

## Why BioSci Toolkit exists – addressing structural gaps

Despite growing investment in STEM, the talent pipeline remains uneven [12,13]. Many students lack clarity on career pathways beyond academia and face limited access to mentors or professional networks. Graduate roles in life sciences are competitive, and inequities persist in the early research career [14,15].

Improving the STEM talent pipeline is not only a matter of fairness; it is essential to ensuring that organisations are robust enough to have sustainable impact. Furthermore, a diverse talent pipeline ensures workforces genuinely reflect the values and experiences of the communities they aim to serve [16,17].

BioSci Toolkit CIC bridges these gaps by providing structured pathways to mentorship, work experience, skills development and a sense of belonging that is often missing in traditional education. A sense of belonging can be defined as “feeling a connection and being part of a group, like a school, workplace or social circle”.[18] [19,20].

Dost and Mazzoli Smith took it one step further and defined belonging in higher education as “feeling part of somewhere an individual can be themselves and feel confident in their personal and social identities, through secure, meaningful and harmonious support in cohesion with other diverse group members and creating ethnically heterogeneous communities and learning areas both on and off the faculty/campus setting [21]. Where there is a sense of belonging, a student flourishes overall, which feeds into academic performance and wellbeing [22].

## Key programmes and activities

### Mentorship and work experience scheme

Through the mentorship scheme, mentors and mentees submit their information via an online form, after which a BioSci team member strategically pairs them based on shared characteristics such as gender, ethnicity and career interests. Mentors are professionals working in industry, the NHS or academia and support students with career planning, application preparation and navigating university life. Mentees on the programme have said it is “…*very helpful to learn about what life after university looks like and helpful in learning about things people don’t tell you about university*”, underscoring the added science and social capital afforded through this expanded professional network.

**Figure 1:**
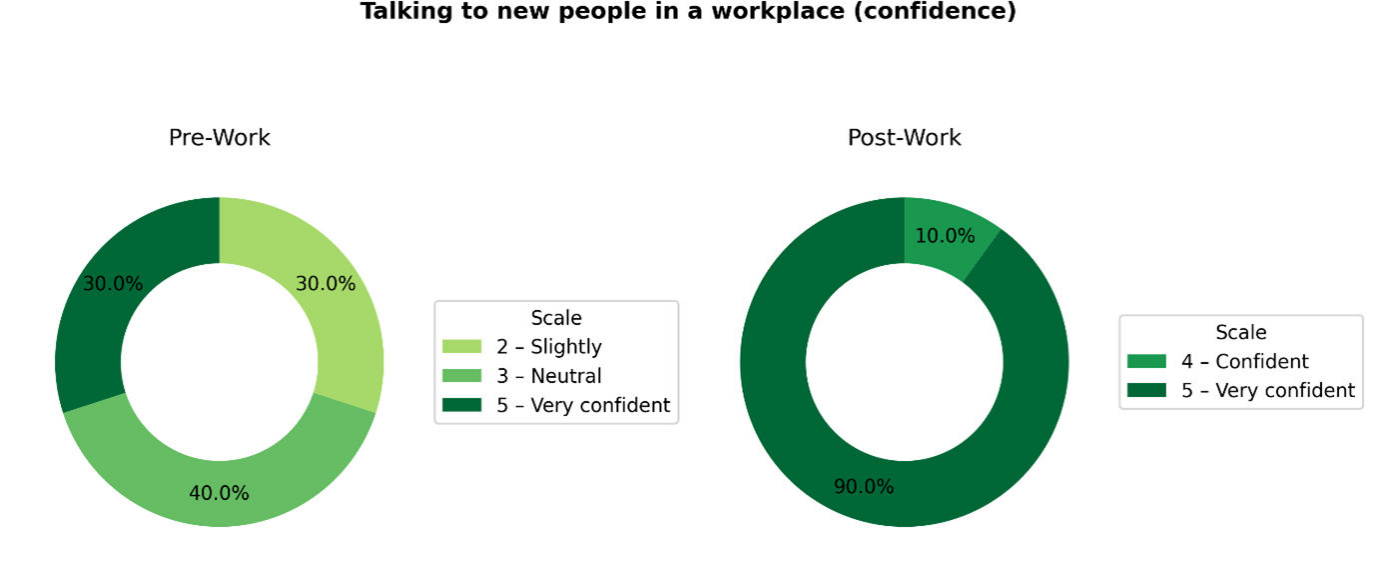
Speaking Confidence Before and After Work Experience. Changes in students’ self-reported confidence in speaking with new people before and after completing work experience at Gilead Sciences, *N* = 10.

BioSci Toolkit CIC has also partnered with organisations such as the biopharma company Gilead Sciences to create opportunities for students to access bioscience career insight days. Our initial pilot involved ten BioSci Toolkit students aged 17–21 visiting Gilead Sciences for three days, attending talks, networking with staff and participating in team-building activities. This experience provided university students with clarity and confidence while giving college students valuable exposure to bioscience career pathways within the pharmaceutical industry. An example of these confidence gains is shown in Figure 1.

By engaging both college and university students, we help to strengthen the STEM talent pipeline, aligning with research showing a positive correlation between science capital and the likelihood of pursuing STEM at A-level, degree level and beyond [23–25].

These two schemes play a crucial role in supporting students’ career development, skills development and confidence, particularly given that many STEM graduates do not pursue STEM-related jobs after university. According to the National Audit Office, only 24% of students who completed STEM courses in England were working in STEM occupations six months after graduation [26]. As identified in the 2016 Wakeham Review of STEM degree provision, stronger communication channels between higher education and industry are needed, as employers are often better positioned than universities to understand the requirements of specific roles. One example highlighted in the review was Energus, an organisation that bridges the gap between universities and employers in the nuclear industry through training and skills programmes. They also emphasised the need to embed soft skills such as presentation delivery, teamwork, report writing and adaptability into degree programmes to enhance work readiness [27].

### School outreach and skills development workshops

Stereotypes about who ‘belongs’ in science are established and reinforced from an early age. Since their inception in the 1980s, Draw-a-Scientist studies have consistently shown that children’s perceptions of scientists are shaped by enduring, outdated biases [28–30]. As writer and activist Marian Wright Edelman aptly said in her speech, “You can't be what you can't see”, highlighting the critical role of representation in shaping aspirations and opportunities. That said, the prospect of ‘being the first’ remains a reality for many, and BioSci Toolkit aims to equip students with the confidence and competence to thrive in these circumstances.

**Figure 2:**
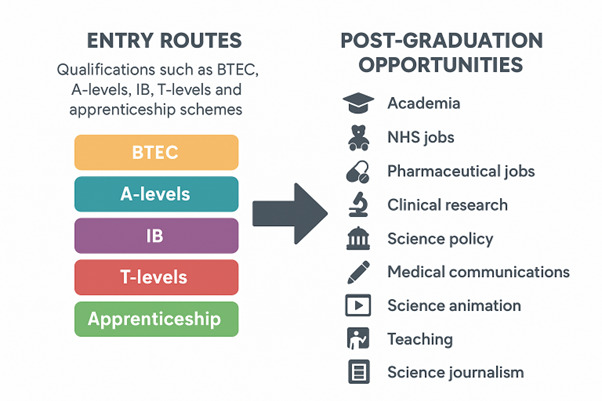
STEM Entry Routes and Career Pathways Entry routes into STEM education and career pathways post-graduation (AI generated image).

BioSci Toolkit’s school outreaches and career talks connect school students with current bioscience students and industry professionals who act as visible role models, raising aspirations and expanding knowledge of career options in sectors and routes to enter those careers. A career advisor from one of our partner schools, Capital City College, said this: “*The resources and knowledge shared to our students encourages them to explore the range of possibilities in the world of bioscience and has helped them to raise their aspirations with careers talks and informative sessions on STEM choices*”. These opportunities are priceless, as there is a shortage of specialist STEM teachers in schools, with fewer STEM graduates entering teaching, and this can lead to a poorer quality teaching experience for students or exposure to learning beyond the classroom [31,32]. Furthermore, within schools, access to quality careers education can vary according to geographical area and school funding, despite being crucial for overcoming issues and raising awareness and understanding of STEM careers, thereby promoting more social inequalities [33,34]. Figure 2 further illustrates the range of STEM entry routes and post-graduation opportunities that the BioSci Toolkit team frequently emphasises to students.

On the website, there are downloadable resources covering lab report planning, CV writing, study skills and support for international students transitioning into UK universities – helping learners balance academic demands with wellbeing and practical life skills.

## Positioning within the UK’s research landscape

Black individuals make up 4% of the UK’s working-age population and 8% of science undergraduates, yet they account for only 0.7% of science professors [35]. Representation of minority ethnic researchers decreases with seniority, with Asian researchers comprising 8.3%, Black researchers 0.6%, those of mixed or multiple ethnicities 1.3%, and other ethnic groups 1.7% of senior positions. Evidence further highlights that individuals of Black Caribbean, Bangladeshi and Pakistani backgrounds are particularly underrepresented [35,36].

While undergraduates from minority ethnic backgrounds in the UK are relatively well represented, their presence declines markedly at more senior academic levels, where the proportion of white scientists correspondingly increases. This pattern has been linked to experiences of isolation, limited access to leadership opportunities, microaggressions and constrained career progression [37–40].

## Future directions and challenges

BioSci Toolkit seeks to transform access to STEM education and careers by scaling regionally and digitally through online mentoring and strategic partnerships with universities and STEM employers. They envision co-developing tailored resources with schools and universities while embedding sustained support for teachers, careers advisers and employers to foster a truly inclusive and diverse talent pipeline. Looking ahead, BioSci Toolkit aims to overcome challenges such as securing long-term funding amid diversity fatigue and sector budget constraints, preserving personalised support at scale, and ensuring that efforts to empower underrepresented communities are systemic and enduring, rather than temporary interventions. Its mission is particularly timely, as the UK educational landscape faces significant challenges, including funding reductions and widespread redundancies among academic and professional staff [41,42].

Moving forward, BioSci Toolkit will evaluate progression and success using a range of metrics, including participant feedback, the proportion of target students pursuing postgraduate studies, and the number entering STEM careers within 3–6 months of graduation. These data will inform continuous improvement, helping to identify and address systemic barriers to inclusion. By highlighting success stories and showcasing diverse role models, particularly in senior STEM positions, BioSci Toolkit aims to create a sustainable pathway that not only supports individual advancement but also contributes to broader structural change within the sector.

BioSci Toolkit CIC may encounter challenges in supporting participants to navigate systemic barriers, particularly in accessing research placements, funding and professional recognition. A constructive approach to addressing these challenges is through sustained collaboration with aligned STEM community partners, including In2Science UK, SISTEMUK, Black in Biomed, Wenite and learned societies such as the Biochemical Society and Physiological Society. By strengthening these partnerships, BioSci Toolkit can amplify opportunities, provide robust mentorship and foster inclusive networks that empower participants. Ultimately, these collaborative efforts not only help individuals overcome barriers but also contribute to creating a more equitable and inclusive STEM ecosystem, inspiring the next generation of scientists to thrive and lead.

## Conclusion

As the UK life sciences sector evolves and grows, creating inclusive, skilled pathways into STEM careers becomes vital. Diversity in STEM can help promote adaptability, drive innovation and help deliver relevant solutions to tackle such global challenges. BioSci Toolkit CIC aims to ensure no one is left behind or missed out as the UK builds its future in science, health and innovation.

**Summary points**

- Persistent structural inequities and unconscious biases continue to limit representation of underrepresented groups in senior life sciences roles, affecting both innovation and public trust.
- Targeted interventions such as mentorship, skills workshops and outreach programmes are critical to sustaining diverse pathways into STEM careers.
- Enhancing social and professional support networks is essential for students lacking familial or community resources to persist through the academic and early-career stages.
- Ensuring the expansion of BioSci Toolkit CIC demands a careful balance between financial sustainability and maintaining bespoke, high-value support for Black, mixed and other underrepresented communities.

## Open Access

This article has been published open access under our Subscribe to Open programme, made possible through the support of our subscribing institutions, learn more here: https://portlandpress.com/pages/open_access_options_and_prices#conditional

## Abbreviations

BTEC: Business and Technology Education Council
CIC: Community Interest Company
IB: International Baccalaureate
NHS: National Health Service
R & D: Research and Development
STEM: science, technology, engineering and maths
T Level: Technically-based qualifications
